# Human epicardial organoids from pluripotent stem cells resemble fetal stage with potential cardiomyocyte- transdifferentiation

**DOI:** 10.1186/s13578-024-01339-w

**Published:** 2025-01-17

**Authors:** Fanwen Wang, Xinle Zou, Huilin Zheng, Tianci Kong, Duanqing Pei

**Affiliations:** 1 College of Life Sciences, Zhejiang University, Hangzhou, China; 2 Laboratory of Cell Fate Control, School of Life Sciences, Westlake University, Hangzhou, China; 3 College of Biological & Chemical Engineering, Zhejiang University of Science and Technology, Hangzhou, China; 4 Westlake Laboratory of Life Sciences and Biomedicine, Hangzhou, China

**Keywords:** Epicardial organoid, Epithelial-mesenchymal transition, Heterogeneity, Paracrine, Epicardial-derived cells

## Abstract

Epicardium, the most outer mesothelium, exerts crucial functions in fetal heart development and adult heart regeneration. Here we use a three-step manipulation of WNT signalling entwined with BMP and RA signalling for generating a self-organized epicardial organoid that highly express with epicardium makers *WT1* and *TCF21* from human embryonic stem cells. After 8-days treatment of TGF-beta following by bFGF, cells enter into epithelium-mesenchymal transition and give rise to smooth muscle cells. Epicardium could also integrate and invade into mouse heart with *SNAI1* expression, and give birth to numerous cardiomyocyte-like cells. Single-cell RNA seq unveils the heterogeneity and multipotency exhibited by epicardium-derived-cells and fetal-like epicardium. Meanwhile, extracellular matrix and growth factors secreted by epicardial organoid mimics the ecology of subepicardial space between the epicardium and cardiomyocytes. As such, this epicardial organoid offers a unique ground for investigating and exploring the potential of epicardium in heart development and regeneration.

**Supplementary Information:**

The online version contains supplementary material available at 10.1186/s13578-024-01339-w.

## Introduction

Epicardium is a mesothelial layer enveloping the outer surface of vertebrate heart. During mouse embryonic development, epicardium emerges from a transient structure called pro-epicardium organ (PEO) at embryonic day (E) 8.5 [1]. PEO cells migrate and differentiate into epicardium and embed myocardium. During development, a subset of epicardial cells detach from epicardium layer, undergo epithelial-mesenchymal transition (EMT) or epithelial to mesenchymal transition, to invade into subepicardium and myocardium layers and give rise to fibroblast, endothelium, smooth muscle cells (SMC) and blood progenitors.

Epicardium has become a focus of investigation, largely due to reports on its role in the innate regeneration of zebrafish heart following injury [2]. While most epicardium goes into dormancy exhibiting limited proliferation ability after birth, some can be reactivated to re-entre into cell-cycle by myocardial infarction (MI) and re-express genes resemble to embryonic epicardium, such as *TBX18* and *RALDH2* [3–7]. Recently, Xia *et al.* identified *ptx3a+/col12a1b +* cells as transiently activated epicardial progenitor cells following cardiac injury in zebrafish, that migrate to the wound and initiate the repair and regeneration processes [8].

Given its purported significance, epicardium has been investigated first in vitro based on human pluripotent stem cells (hPSCs) [9] using BMP activator and WNT inhibitor. It has been subsequently shown that WNT alone is sufficient to constrain terminal-differentiated cells into epicardium but not cardiomyocytes (CMs) [10]. Furthermore, epicardium could be generated within 7 days by inhibiting WNT following by activating BMP4, Retinoic acid (RA) and vascular endothelial growth factor (VEGF) [11]. Meier and colleagues [12] reported the generation of epicardioid which has myocardium embedded in epicardium layer as a model to investigate the interactions between epicardium and CMs. However, due to the non-renewability of CMs, the heart injury in adult mammals may lead to grave consequences. Inducing the original epicardium or implanted epicardium into CMs are alternatives for heart injury treatment. Unfortunately, it is not well-established for criterion of factors triggering epicardial transdifferentiation in vivo, although thymosin beta 4 and VEGF has been reported [13, 14]. In addition, conversion of exogenous epicardium into myocardium is inefficient or absent in mammals such as mice and pigs [15] and may come up to approximately 15% in salamander [16]. Low efficiency limits its application.

In this study, we describe a process to generate epicardial organoids that continuously expresses of epicardium markers *WT1*, *TCF21* and *TBX18*. The organoid shows TGF-beta and bFGF-induced EMT functions and transdifferentiates into SMC. Combined with single-cell RNA sequencing (scRNA-seq) and mass spectrum, we found epicardium produce different extracellular matrix (ECM) mRNA and the inner side of chamber accumulates ECM such as collagens and fibronectin. More importantly, cells of this epicardial organoid can integrate with adult mouse heart and migrate into the inner parts of heart which is similar with the process of PEO migration onto heart tube. These derived cells showed myocardial morphological similarity. Taking together, these results provide evidence that these epicardial organoids recapitulate features of human fetal epicardium and manifest potential in heart regeneration.

## Methods

### Generation of 2D epicardium and 3D epicardial organoid

Human embryonic stem cells (hESCs) and home-made UiPSC were cultured in mTeSR1 medium (Stemcell Technologies, #85850) on Matrigel (Corning, #354230, GFR, LEDV-free) coated plates and passaged using either TrypLE Express Enzyme (Thermo Fisher, #12563029) or 0.5 mM EDTA-DPBS (Sigma-Aldrich, #E6758) every 2 ~ 4 days at 70% ~ 90% confluency. Cells were routinely tested for absence of Mycoplasma contaminations. All cell lines were cultured at 37 °C in a humidified atmosphere containing 5% CO_2_. hPSCs were de-attached using Accutase (Stemcell Technologies, #07920) and resuspended in mTeSR1 containing 10 µM ROCKi Y-27,632 (TargetMol, T1725). 20,000 cells in 150 µL medium were seeded into Ultra-Low-Attachment U-bottom 96-well plates (Corning #7007 or Thermo Fisher #174929) on which was recorded as day − 1 and left settled for 24 h. For 2D differentiation, 150,000 cells were seeded on a Matrigel-coated 24 well. On day 0, medium was changed with CDM [17] containing 30 ng/mL bFGF (Peprotech, 100-18B), 3 ng/mL BMP4 (R&D systems, 314-BP-500), 10 µM CHIR99021 (Tocirs, 4423 or Selleck, s1263), 5 µM LY294002 (Selleck, S1105) and 50 ng/mL Activin A (Peprotech, 120-14-1000). The composition of CDM was 50% IMDM (Gibco, 12440-053) plus 50% F12 NUT-MIX (Gibco, 11765-054) supplemented with 15 µg/mL transferrin (Sigma-Aldrich, T8158), 450 µM monothioglycerol (Sigma-Aldrich, M6145) and 5 mg/mL BSA (Sigma-Aldrich, SRE0098). After a 36 h ~ 40 h nourishment, embryonic bodies were then incubated for 2 days with CDM medium containing 3 ng/mL BMP4, 5 µM IWP2 (Selleck, S7085), 5 µM SB431542 (Selleck, S1067), 1 µM BMS (Sigma-Aldrich, SML1149) and 0.5 µM PD-173,074 (AbMole, M1875). From day 3.5 to day 6.5, spheroids were cultured in CDM medium containing 10 ng/mL BMP4, 5 µM CHIR 99,021 and 1 µM RA (Selleck, S1653) with a refreshment on day 5. Subsequently, CDM medium containing 10 µg/mL insulin was used for another 3 days. For maintaining epicardium, CDM medium containing 10 µg/mL insulin (Selleck, S6955) and 5 µM SB-431,542 was employed from day 9.5 onwards and refresh organoids every 3 ~ 4 days. Chamber would be visible on day 9.5 from where additional investigation could be performed.

For study the EMT function, Organoids from day 9.5 were treated with 5 ng/mL TGF-beta1 (R&D systems, 240-B) following 10 ng/mL bFGF (Peprotech, 100-18B) for 4 days each and tested at day17.5.

### Cryosectioning

4% PFA-fixed organoids were cryoprotected with 30% sucrose in PB for 1 ~ 2 days at 4℃ until sinking to the bottom of the tube and washed twice with PBS before embedded in O.C.T. (Sakura, #4583). Embedded organoids were transferred onto the O.C.T.-coated tray on a Leica cryostat at -22℃ ~ -20℃. 10 μm sections were collected on Ultra Plus slides (CITOTEST, # 188105) and kept at -80℃ until immunostaining. O.C.T. was removed by washing with PBS before continuing with the immunostaining protocol.

### Immunostaining

Cryosections were fixed with 4% PFA (Beyotime, #P0099) and permeabilized with 0.2% Triton X-100 (Sigma-Aldrich, #T9284) in block solution (Beyotime, #P0102) for 15 min each. Sections were then incubated in block solution for 1 h. The primary antibody was subsequently applied in primary antibody dilution medium (Beyotime, #P0103) overnight at 4℃ and subjected to the secondary antibody in secondary antibody dilution medium (Beyotime, #P0108) for 1 h.

Antibodies employed in this study are as follows: Brachyury (R&D Systems, AF2085-SP), AFP (Santa Cruz, SC130302), α-SMA (Abcam, ab7817), CASP3 (Cell Signaling Technology, 9661), Calponin (Sigma-Aldrich, C2687), CDH1 (CST, 14472 S), CDH5 (CST, D87F2), CDX2 (Abcam, ab76541), HNF-4α (Santa Cruz, sc-6557), HNA (Chemicon, MAB1281), Ki-67 (BD Bioscience, 556003), SNAI1 (Abnova, H00006615-B02P), TNNT2 (Abcam, ab8295), VIM (Cell Signaling Technology, 5741 S), WT1 (Abcam, ab89901 or CST, 83535T), ZO-1 (CST, 5406 S). Slides were mounted using fluorescence mounting medium (Abcam, #ab104139). Three times wash with PBS or PBS/0.1% Tween20 were required between different reagents treatment. Slides were kept at -20℃ until imaging. All the incubations were performed at room temperature unless otherwise specified. Images from more than 3 organoids each group were collected with inverted confocal microscopes (Zeiss, LSM900 or LSM800). Images were processed by Fiji/ImageJ (v2.0, https://imagej.net/Fiji.html ) and Zen 3.4 (blue edition, https://portal.zeiss.com/download-center/softwares/mic ).

### Mass spectrometry

Organoids were washed in DPBS before collecting the inner fluid using a pulled glass microcapillary attached to filter and tubing as previously described with some modifications [18]. Samples were centrifuged at 250 g for 5 min to remove dead cells and debris. Polyacrylamide gel slices (1 ~ 2 mm) containing the purified proteins were prepared for mass spectrometric analysis. The excised protein gel pieces were destained with 50% v/v acetonitrile and 50 mM ammonium bicarbonate, reduced with 10 mM DTT, and alkylated with 55 mM iodoacetamide. After alkylation, proteins were digested with 200 ng/µL Trypsin (GenDEPOT, #T9600-025) for 16 ~ 18 h at 37 °C. The resulting peptides were extracted in 2% v/v formic acid, 100% v/v acetonitrile. The digest was analyzed by nanoscale capillary LC-MS/MS using an Ultimate U3000 HPLC (Exploris 480) to deliver a flow of approximately 300 nL/min. A C18 Acclaim PepMap100 5 μm, 100 μm x 20 mm nanoViper (Thermo Scientific Dionex, San Jose, USA), trapped the peptides prior to separation on a C18 Acclaim PepMap100 3 μm, 75 μm x 250 mm nanoViper (ThermoScientific Dionex, San Jose, USA). Peptides were eluted with a 60 min gradient of acetonitrile (2–80%). The analytical column outlet was directly interfaced via a nano-flow electrospray ionisation source, with a hybrid quadrupole orbitrap mass spectrometer (Q-Exactive Plus Orbitrap, ThermoScientific, San Jose, USA). Data dependent analysis was carried out, using a resolution of 30,000 for the full MS spectrum, followed by ten MS/MS spectra. MS spectra were collected over an m/z range of 300–2000. MS/MS scans were collected using a threshold energy of 27 for higher energy collisional dissociation (HCD). LC-MS/MS data were then searched against a protein database (UniProt KB) using the Mascot search engine programme (Matrix Science, UK). Database search parameters were set with a precursor tolerance of 10ppm and a fragment ion mass tolerance of 0.8 Da. One missed enzyme cleavage was allowed and variable modifications for oxidized methionine, carbamidomethyl cysteine, pyroglutamic acid, phosphorylated serine, threonine and tyrosine, and methyl arginine were included.

### Transmission electron microscopy

Organoids were performed with transmission electron microscope (TEM) Thermofisher Talos 120. Briefly, Samples were fixed with 2.5% glutaraldehyde and 2% PFA in 0.1 M PB buffer (pH 7.4) at 4℃ overnight. After washed with PB buffer, organoids were post fixed with 1% osmium in 0.1 M PB buffer on ice for 60 min and washed by PB buffer. Samples were then stained with 1% uranyl acetate in ddH_2_O at 4℃ overnight. After a gradual-dehydration with a graded series of ethanol (50%, 70%, 90%, 95%, and twice in 100%), they were infiltrated with and embedded in EPON12 resin with a 48 h-polymerization at 60 °C, and cut into 70-nm-thick ultrathin sections which were then post stained with uranyl acetate and Sato’s Lead. Sections were then ready for examination at 80 kV using Talos 120.

### RNA isolation, qRT–PCR and bulk RNA-seq

Total RNA was extracted using the RNA-easy Isolation Reagent (Vazyme, #701) according to manufacturer instructions and quantified using a Nanodrop spectrophotometer (Thermo Fisher Scientific, Waltham). For quantitative PCR, cDNAs were synthesized with ReverTra Ace (Toyobo) and oligo-dT (Takara), and levels of the mRNA were analysed by qRT-PCR with ChamQ SYBR qPCR Master Mix (Vazyme). The qRT-PCR primers used in this study are as follows: GAPDH-qF, GCACCGTCAAGGCTGAGAAC, GAPDH-qR, CGCCCCACTTGATTTTGG, WT1-qF, CCAATACAGAATACACACG-CAC, WT1-qR, CATCTGTAAGTGGGACAGCTTA, TCF21-qF, AGG CAG ATC CTG GCT AAC GAC AAA, TCF21-qR, TCC AGG TAC CAA ACT CCA AGG TCA, TBX18 -qF, TTA ACC TTG TCC GTC TGC CTG AGT, TBX18 -qR, GTA ATGGGCTTTGGCCTTTGCACT, TWIST1-qF, GGAGTCCGCAGTCTTACGAG, TWIST1-qR, TCTGGAGGACCTGG-TAGAGG, SNAI1-qF, TTT CTG GTT CTG TGT CCT CTG CCT, SNAI1-qR, TTC CCA GTG AGT CTG TCA GCC TTT, Vimentin-qF, CCGGAGACAGGTGCAGTCCCT, Vimentin-qR, TCATCCTGCAGGCGGCCAAT, SMTN-qF, AGC ACC ATG ATG CAA ACC AAG ACC, SMTN-qR, TCT GCG CCT TCA TCA GCT CTT TCT, ZO1_qF, TGCCATTACACGGTCCTCTG, ZO1_qR GGTTCTGCCTCATCATTTCCTC, OCLN_qF, ACTGCTCAGTCTTCTGGATC, OCLN_qR, TGTCATACCTGTCCATCTTTCTTC, CLDN1_qF, TTCTCGCCTTCCTGGGATG, CLDN1_qR, GCTCAGATTCAGCAAGGAGTCA, CDH1_qF, GCCTCCTGAAAAGAGAGTGGAAG, CDH1_qR, TGGCAGTGTCTCTCCAAATCCG. For library constructions, VAHTS mRNA-seq V3 Library Prep Kit for Illumina (Vazyme, #NR611) was used. Library was further tested using an Aguilent 2100 Bioanalyzer followed by RNA sequencing with Illumina Novaseq PE150 platform. RNA-seq data was analyzed by DEseq2, GO.db, Mfuzz, Phantasus 1.11.0 (artyomovlab.wustl.edu/phantasus) and ToppGene Suite (http://toppgene.cchmc.org*).*

### Single-cell (sc)RNA-seq and bioinformatic analysis

Organoids were dissociated into single cells using the STEMdiff Cardiomyocyte Dissociation Kit (Stemcell Technologies, #05025). Cells were counted and checked for viability using a hemocytometer (Thermo Fisher, Countess 3) before preparing library. After dissociation, samples were processed for scRNA-seq with a targeted cell recovery of 20,000. To generate Gel Bead-In-EMulsions (GEMs) and single-cell sequencing libraries, the Chromium Next GEM Single Cell 3′ Library & Gel Bead kit v3.1 (10x Genomics, 1000128), Chromium Single Cell G Chip kit (10x Genomics, 1000127) and Chromium i7 Multiplex kit v2 (10x Genomics, 120262) were used. Quality control of cDNA was performed on a Bioanalyzer (Agilent) using a high-sensitivity DNA kit (Agilent, 5067 − 4626). Library quantification was performed with the KAPA quantification kit (KAPA Biosystems, KK4824). Libraries were pooled and sequenced using a NovaSeq S1 flow cell (Illumina) with 150-base pair (bp) paired-end reads with 28 cycles for read 1, 91 cycles for read 2, 8 cycles for i7 and with a read depth of at least 25,000–30,000 paired-end reads per cell.

Single-cell gene counting matrix was generated by performing sample demultiplexing and barcode processing using Cell Ranger pipeline (v6.1.1). Further analysis was ran using R statistical programming language (Rstudio v4.3.3, https://www.rstudio.com ). The count data matrix was read into R and used to construct a Seurat object (v5.1.0). Data were filtered and normalized. Briefly, cells with fewer than 3 expressed genes in the sample, cells with fewer than 300 but more than 9,000 expressed genes, and cells with mitochondrial genes greater than 25% of the total number of unique molecular identifiers (UMIs) and ribosomal genes greater than 50% of the total number of UMIs were excluded. High variable genes were identify identified for helping annotate clusters by employing Seurat data processing pipeline. Cellular dynamics were inferred based on the kinetics of gene expression using RNA velocity.

### Generation of reporter cell lines

Generally, hPSCs were transfected with pB-CAG-tdTomato and PiggyBac transposase by using Effectene Transfection Reagent (QIAGEN #301425) according to manufacturer instructions. While cell growth reached 50% confluency, cells were de-attached by Accutase and seeded into 96-well-plate at 1 cell per well. Once single colonies were macroscopic after 4 ~ 7days, the colonies with fluorescence were picked and transferred into 24-well-plates under microscope.

### Transplantation of epicardial organoid

NOD-SCID mouse were perchased form Jiangsu Jicuiyaokang and genotyped with Prkdc. Mouse were examined with heart function by using Vevo 3100 (FUJIFILM VisualSonics) and electrocardiograph (ECG, Kent/Biopac CODA/MP160) before and after transplantation. The data of ultrasound and ECG were then analyzed by using Vevo LAB (FUJIFILM VisualSonics, Inc. https://www.visualsonics.com) and AcqKnowledge (BIOPAC Systems, Inc. https://www.biopac.com/support/biopac-software-updates/), respectively.

For surgery, mice were anesthetizing with 1.2% Avertin at a dosage of 240 mg/kg. Intratracheal intubation connecting to a ventilator was performed and the pectoral muscle and ribs were cut to expose the heart. The pericardium was pulled away and epicardium lined outside the right ventricular heart was tear to make a transplant entrance. Two or three organoids were placed onto the subepicardium space. The ribs and skin were then closed with suitable sutures. Mouse were then treated with 100 µL of 5 mg/kg Cyclosporine (Targetmol T6459) and 2.5 mg/kg Methylprednisolone succinate (Targetmol T22367) subcutaneously every day for at least 2 weeks. Mouse were then sacrificed for further investigation.

### Whole-organ imaging

Mouse were anesthetizing with 1.2% Avertin at a dose of 250 ~ 300 mg/kg before thoracotomy. 30 mM KCl and 0.9% NaCl were then sequentially injected into the left ventricle to remove circulating blood. Heart was then harvested and fixed in 4% PFA at 4 °C for 24 h. After washing using PBS, pigment was decolorized by specific solution (Nuohai Life Science, NH-230701-Dep) for 24 h. For tissue clearing, Enhanced Tissue Clearing Kit (Nuohai Life Science, CR-230701-S) was employed. Briefly, tissue was defatted in solution A for 24 h, stained for nuclei for 5 days, and incubated with the primary and secondary antibodies for 4 ~ 7 days of each. Solution B is used for refractive index matching for 2 days. After embedding in low-melting-point agarose, hearts were imaged using a laser scanning microscope (Nuohai Life Science, LS18) in imaging solution. The images are then combined and stitched before being processed with Imaris (Oxford Instruments, v10.0, https://imaris.oxinst.com/ ).

### Quantification and statistical analysis

All analyses were performed using GraphPad Prism (v9.0.0) (https://www.graphpad.com ) software and all raw data was collected in Microsoft Excel. All data presented a normal distribution. Statistical significance was evaluated with a standard unpaired Student t-test (2-tailed; *p < 0.05, **p < 0.01, ***p < 0.001) when appropriate. For multiple-comparison analysis, one-way ANOVA with the Tukey’s or Dunnett’s post-test correction was applied when appropriate (*p < 0.05, **p < 0.01, ***p < 0.001). All data are presented as Mean ± SEM and represent a minimum of 3 independent experiments with at least 3 technical replicates unless otherwise stated. All micrograph images are representative of at least 3 independent experiments per condition/marker and calcium transient graphs are representative of 3 independent experiments.

## Results

### Generation of epicardial organoid from hPSCs

**Fig. 1:**
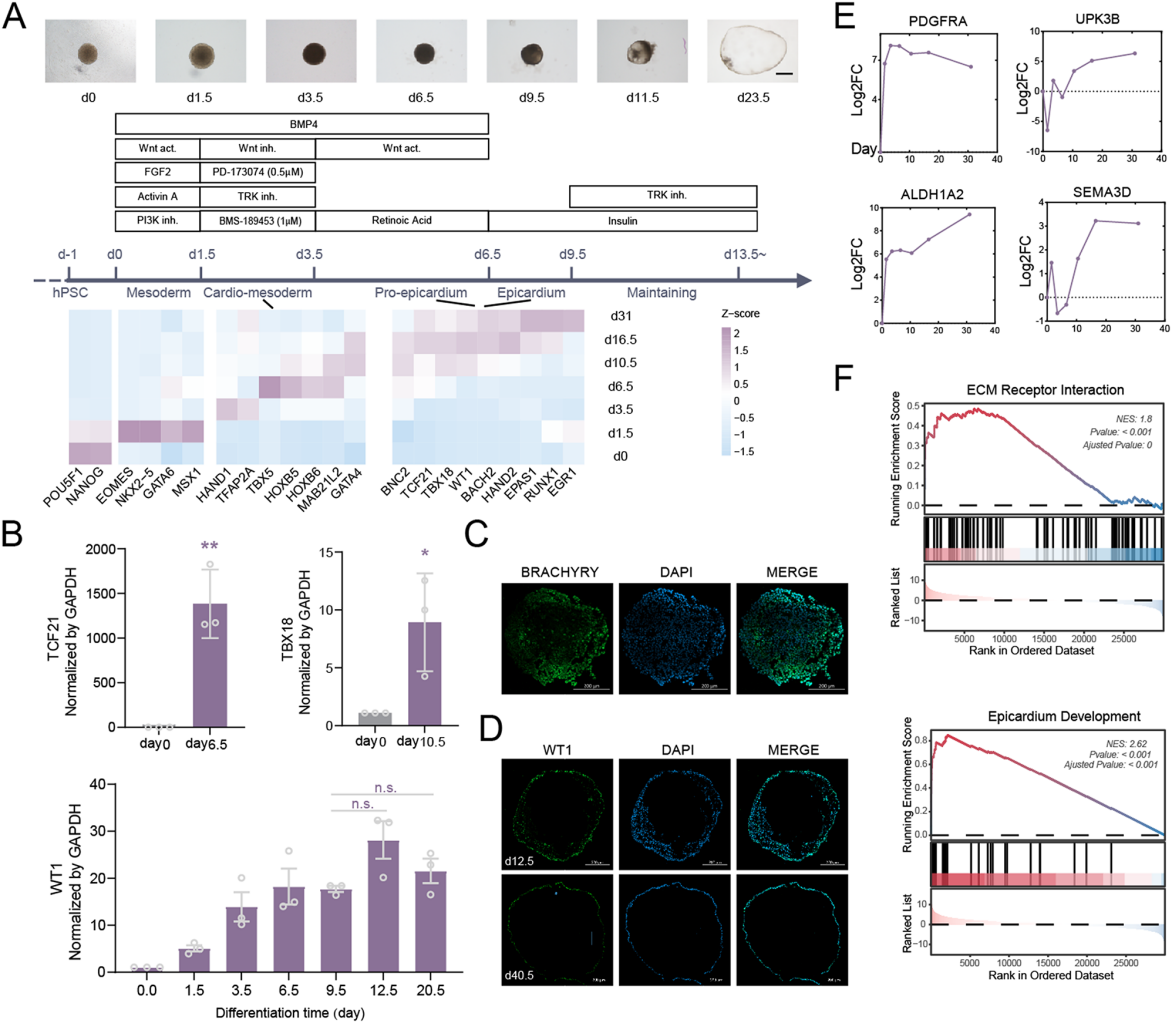
Generation of epicardial organoid from hPSCs. (**A**) Up, bright field images captured with time course showed gradually enlarged chamber. Middle, scheme of the protocol used to differentiate hPSCs toward the epicardium lineage highlighting four main stages of development: (i) mesoderm induction, (ii) cardiac progenitor cells canalization, (iii) PE induction, (iv) Epicardium specification. Key chemicals used in each step are list under the arrow. Down, heatmap of representative genes on time course bulk RNA sequencing at d0, d1.5, d3.5, d6.5, d10.5, d16.5 and d31 with two replicates each timepoint. (**B**) Quantification qRT-PCR analysis on epicardium markers *TCF21* at d6.5, TBX18 at d10.5 and *WT1* at critical knots. Immunofluorescence of mesodermal marker BRACHYURY at d1.5 (**C**) and epicardium marker WT1 at d12.5 and d40.5 (**D**). (**E**) Expression level (log2FC) of other reported epicardium markers, *PDGFRA*,* SEMA3D*,* UPK3B* and *ALDH1A2*. (**F**) GSEA score of enriched genes at d10.5 showed ECM receptor pathways and epicardium development are specifically activated. Scale bar, 500 μm. Significance analysis uses a standard unpaired Student t-test (2-tailed; *p < 0.05, **p < 0.01, ***p < 0.001, n.s. no significance)

To generate epicardioids structurally resembling those in vivo, we design a strategy that can induce hPSCs towards epicardium lineage in four sequential stages: (i) induction of mesodermal cells by modulation BMP and WNT pathways, (ii) canalization for cardiac progenitors, (iii) specification of PEO with RA, (iv) terminally differentiation and maintaining of epicardium using TRK inhibitor (Fig. 1A middle). Consistently, we show that commonly used epicardium markers *TCF21*,* WT1*, and *TBX18* can be first detected on d6.5 by qRT-PCR analysis (Fig. 1B, Additional file 1). As expected, mesoderm markers BRACHYRY and *EOMES* are highly expressed at d1.5 with immunofluorescence and bulk RNA seq (Fig. 1C and A down, Additional file 2). The stable epicardioids continue to express *WT1* until d40 (not tested beyond this time points) (Fig. 1D). From bulk RNA seq data, we show that as differentiation progresses, pluripotency genes *POU5F1* and *NANOG* were gradually silenced accompanied by the increase of cardiogenic precursor markers such as *GATA6*,* HAND1*, and *TBX5* (Fig. 1A down). How human PEO emerges and develops into epicardium remains unknown due to the lack of embryo samples less than four weeks post conception [19]. Besides, markers expressed begin at PEO stage, such as *WT1*, *TCF21*, *TBX18*, *SEMA3D* and *UPK3B*, would sustain throughout the embryonic stage, that is PE and epicardium share the same markers. In this organoid, we observed some less common epicardium-fate marker genes that show an increasing trend with differentiation, such as *SEMA3D*,* ALDH1A2*,* UPK3B* [20], *PDGFRA* (Fig. 1E) as an evidence of epicardium development. At d10.5, bulk RNA data indicate that the enriched genes are most related to epicardial development and ECM pathways during the maintenance culture phase (Fig. 1F).

**Fig. 2:**
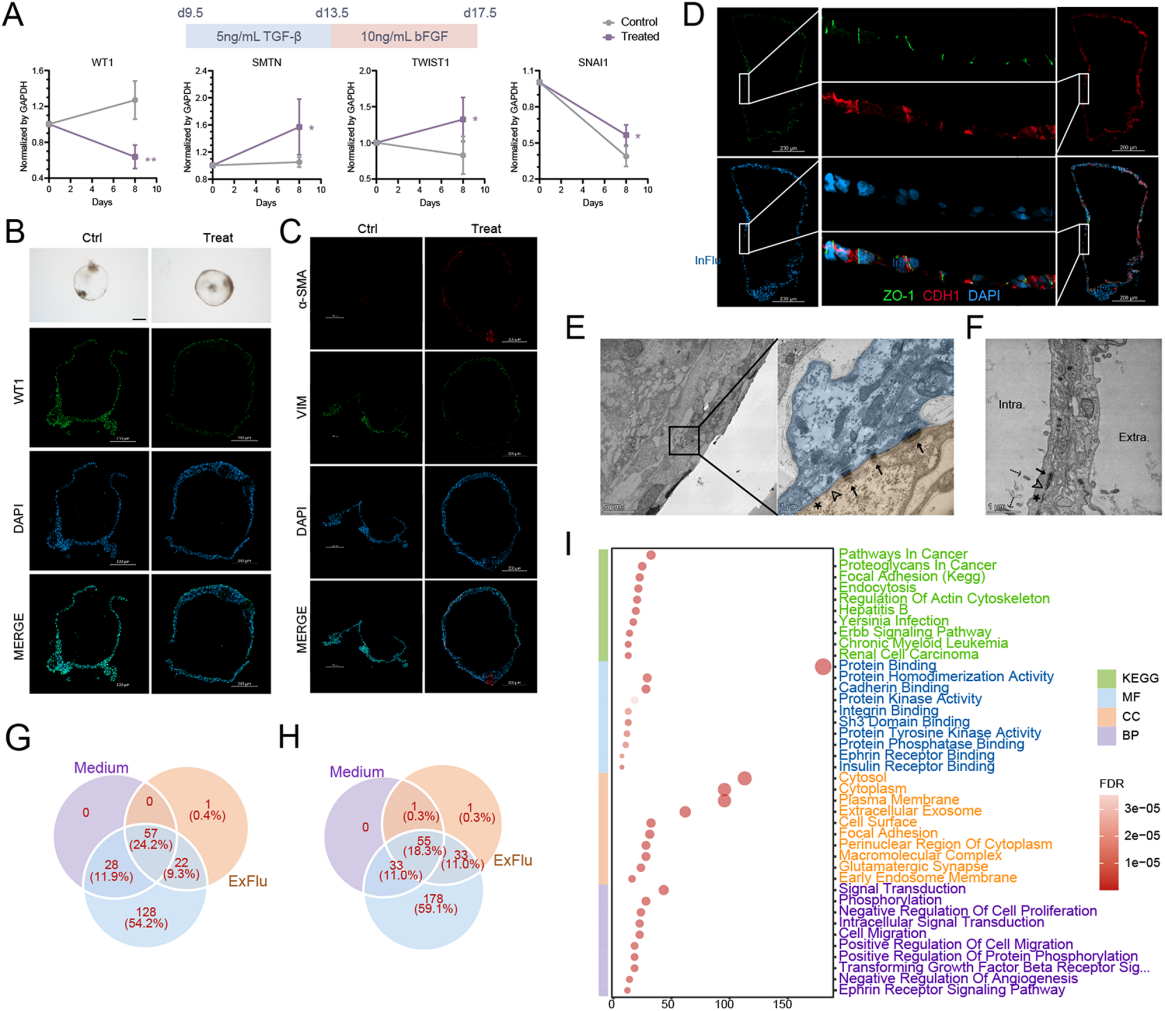
Epicardial organoid underwent EMT to give rise to SMCs and supported with paracrine factors. (**A**) Scheme of the protocol for inducing EMT and qRT-PCR analysis of *WT1*, SMC marker *SMTN* and EMT marker *TWIST1* and *SNAI1*.Bright field images of organoid at 17.5 with or without treatment (**B**, upper, scale bar 500 μm). Immunofluorescence of WT1 (**B**, lower), mesenchymal marker VIM and SMC marker α-SMA at d17.5 (**C**) showed organoid can transit from epithelium to mesenchymal type leading to increase of SMCs. (**D**) Polarity validation by staining ZO-1 (marker of epicardium tight junction) and CDH1 (marker of epicardium adherens junction). (**E**) and (**F**) Transmission electron microscopy image of epicardial organoid at day 108. Desmosomes (arrow), adherens junctions (arrowhead), tight junctions (asterisks) of epicardium, ciliary tract (dash arrow) of EC. Venn diagram of ECM (**G**) and GF (**H**) by comparing fresh culturing medium, inner-organoid fluid (InFlu) and extra-organoid fluid (ExFlu) enriched by mass spectrum. (**I**) KEGG and GO analysis of GFs secreted by epicardial organoid. Significance analysis uses a standard unpaired Student t-test (2-tailed; *p < 0.05, **p < 0.01, ***p < 0.001)

Structure between adjacent epicardium consists of tight junction (TJ), adherens junction (AJ), gap junction (GJ) and desmosome which serves for maintaining permeability and cell polarity. Here, we noticed the universal expression of tight junction scaffolding protein zonula occludens-1 (ZO-1, *TJP1*) and adherens molecule CDH1 on the lateral cell membrane (Fig. 2D). These interactions were also be observed by TEM as early as day 17 (not examined at earlier time points) as shown in Fig. 2E.

During organogenesis, heart, lungs and intestines generate lumen space for rapid substance exchange. Organoids, in which most of the cells are epithelial, are prone to lumen formation [21–23]. This epicardial organoid can also form chamber which typically appears at d9 ~ d11 as observed in the bright-field images, and grows significantly until d20 (Fig. 1A, Supplementary Fig. 1A). Mechanistically, lumens can be classified into cavitation caused by central cell apoptosis or expansion by separation of adjacent cell membranes [24]. During which, primary cilia and mechano-sensitive ion channels such as Piezo1 [25, 26], TRPP2 (also known Pkd2) and Trpv4 [27], as well as actin network connecting F-actin, integrin and cadherin, are the three critical elements involved. For heart development, mechanisms underlying subepicardium lumen formation remain unknown. We also measured apoptosis as epicardioids and show that genes involved in apoptosis including *CASP3*,* BIRC5*,* FAS*,* BAX* are expressed at higher levels at d0 and d1.5, then decrease afterwards. Apoptosis is detectable in epicardial organoid constantly under relatively lower level, but remains constant during chamber formation (Supplementary Fig. 1D, 1E). These results suggest that chamber formation may be independent of apoptosis.

In order to test the universality of epicardial organoid induction program, we also examined the H1 cell line and in-house UiPSCs, which showing similar repeatability with high expression of epicardium markers (Supplementary Fig. 1B, 1C), albeit with some differences in organoid size (Supplementary Fig. 1A). Hereafter, detailed characterizations were proceeded by using H9 cell line.

### Epicardium can undergo EMT and generate SMCs

EMT has been observed in epicardium of human, mouse and chick embryo heart [28–34]. To induce EMT during epicardioid generation, we change culture medium at d9.5 with 5 ng/mL TGF-β for 4 days followed by 10 ng/mL bFGF for 4 days (Fig. 2A). Notably, TGF-β inhibitor SB-431,542 was added into the maintaining culture medium giving that epicardium would spontaneously undergo EMT in the absence of SB-431,542 [35]. In this study, SB-431,542 was not removed during the induction of EMT. While there is no significant change in the size of the organoids, those treated with TGF-β and bFGF exhibit a turbid appearance, indicating cell proliferation (Fig. 2B). Additionally, compared to the control group, the treated group shows a significant decrease of *WT1* (0.64 ± 0.13 vs. 1.27 ± 0.21, *p* = 0.002 **), a significant increase in the classical EMT markers *TWIST1* and *SNAI1* (1.32 ± 0.31 vs. 0.83 ± 0.26, *p* = 0.049 *; 0.57 ± 0.08 vs. 0.39 ± 0.08, *p* = 0.025 *), and a significant increase in the smooth muscle marker *SMTN* (1.57 ± 0.41 vs. 1.05 ± 0.08, *p* = 0.048 *) (Fig. 2A). Thus, treatment with TGF-β followed by bFGF induces EMT in epicardial organoids and leads to smooth muscle generation (Fig. 2C). Moreover, there is a significant decrease in tight junction marker *CLDN1* (0.52 ± 0.19, *p* = 0.0096 **), *OCLN* (0.65 ± 0.02, *p* < 0.0001 ****) and adherens junction marker *CDH1* (0.61 ± 0.05, *p* = 0.0002 ***) when EMT was induced, whereas a subtle reduction in *TJP1* (0.87 ± 0.08, *p* = 0.0562) (Supplementary Fig. 1F, Additional file 1). As the immunofluorescence shows, CDH1 only located in the inner single layer of organoid, and ZO-1 was found in these CDH1^+^ cells accounting for no more than two thirds (Supplementary Fig. 1G). Cell junctions were reduced when EMT was stimulated. These results suggest that the epicardioids are capable of responding to EMT signals and generate smooth muscle cells.

### Epicardium secrets ECM present in human subepicardium

Epicardium provides paracrine factors for myocardium to ensure CMs maturation and repair. Underneath the epicardium, there is a gelatinous space called subepicardium (or subepicardial space), highly enriched with hydrated ECM, including collagens I, IV, V, VI and fibronectin, flectin, fibulin-2, GP68, laminin, proteoglycans, vitronectin, fibrillin-2, elastin and tenascin-X [36] synthesized and secreted by epicardium mostly, as well as growth factors (GFs) such as fibroblast growth factor (FGF) and VEGF. We wish to determine the proteome secreted from epicardioids by mass spectrometry. The proteins identified can be classified into either ECM or GF, among others and we show that 151 ECM molecules are secreted by epicardial organoid (Fig. 2G, Additional file 3). Among them, CRELD1 is the only one present in the extra-organoid fluid. CRELD1 can be expressed by many cell types, such as epicardium, endocardium, fibroblasts and CMs. The gene was translated in endoplasmic reticulum following glycosylation modifications in the Golgi apparatus, and finally anchored in the cell membrane or excreted into ECM. The protein sequence contains two EGF-like domains and two CXXC motifs, which enable binding to cell adhesion molecules and integrins, as well as inducing histone H3K4 methylation and H3K36 demethylation, participating in cell-cell adhesion and epigenetic regulation [37]. Loss-of-function experiments have demonstrated that CRELD1 plays a role in the formation of septa and valves during early embryonic development [38], and contributes to myocardial trabeculation formation and ECM remodeling in later stages [39]. However, since CRELD1 produced by epicardial organoids was secreted outward the chamber, it may not be related to production of other components of the heart, such as the myocardium. Given that Bonaguro *et al* [40] reported that CRELD1 can regulate T-cell proliferation through the WNT pathway, and considering that immune cells are widely distributed in the heart while T cells are present in relatively low numbers [41], we hypothesize that CRELD1 secreted outward by epicardial organoids may be involved in pathogen defense in early fetal stages. Additionally, CRELD1 may serve a lubricating function by remodeling the ECM in the pericardial cavity, located between the epicardium and the parietal pericardium.

There are 22 extracellular matrices existing both in the inner and extra-fluid which are more associated with extracellular exosome, region and space according to GO analysis. The 128 matrix proteins that only shown appearance in the inner-fluid are more likely to be involved in maintenance of structural and electrophysiological characteristics of CMs except the three mentioned above. Moreover, collagens such as COL4A5, COL4A6, COL9A3 and COL22A1 are the most abundant ECM which occupy more than nine out of ten of the cardiac ECM in vivo. The abundance of IV collagen hints the existence of basement membrane. Epicardium specific collagen COL9A3 and COL11A1 [42] was found widely expressed in accord with single-cell transcriptome analysis. As for GFs, we found 212 GFs organoid can secret including TGFB2, IGFBP3 involved in TGF signaling, angiogenesis and focal adhesion (Fig. 2H and I, Additional file 3). These results validate epicardial organoid as in vitro copies of human epicardium based on ECM and GF profiles.

**Fig. 3:**
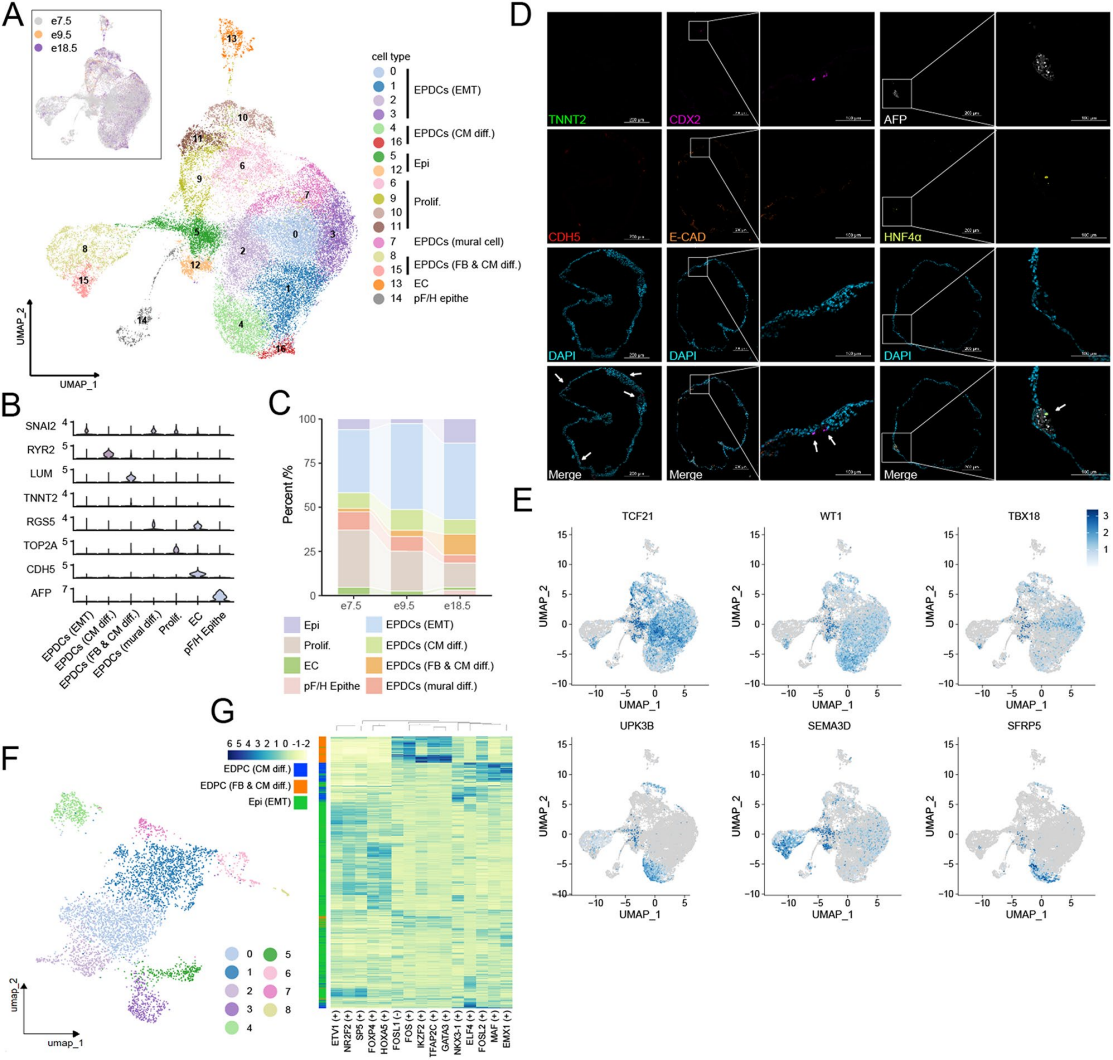
Epicardial organoid with inner heterogeneity. (**A**) UMAP dimensional reduction plot showing the 17 cell clusters obtained by scRNA-seq at d7.5, d9.5 and d18.5; main cell types are annotated. (**B**) Violin plots showing the expression levels of different cell type markers. (**C**) Stream flow plot for cell number percent of different cell type. (**D**) Immunofluorescence label for TNNT2 (CM), CDH5 (EC), CDX2, AFP, and HNF4α (pF/H Epithe). (**E**) Feature plot of canonical epicardial markers. (**F**) Nine subclusters analyzed from c5 and c12. (**G**) Heatmap of differential TF sets between EPDC (CM diff.), EPDC (CM diff.) and Epi (EMT)

### ScRNA-seq profiling of epicardioids

We further interrogate the transcriptomic landscape of epicardioids at d7.5, d9.5 and d18.5 by scRNA-seq. We use unsupervised clustering to identify 17 cell clusters. According to gene expression patterns combined with RNA velocity analysis (Supplementary Fig. 2A), we designate these 49,716 cells into 8 types (Fig. 3A) which revealed strong associations with intercellular junctions such as TJ, AJ, and ECM (Supplementary Fig. 2B). Except for a few endocardium (EC, cluster 13: *CDH5*,* PECAM1*,* TIE1*,* TEK*. 1.6%) and posterior foregut or hepatic epithelium (pF/H Epithe, cluster 14: *AFP*,* HNF4A*,* FOXA2.* 3.08%), all the others are epicardium and its progenitors as well as derivations comprising 95.32% of the total (Fig. 3A-D) expressing pan epicardial markers (*TCF21*, *WT1*,* TBX18*,* SEMA3D)* among which *WT1*^*+*^*TCF21*^*+*^*TBX18*^*+*^ cells were shown to be restricted to the epicardial layer of the ventricular walls [43]. Among them, *UPK3B* and *SFRP5* were enriched in c4, while *SEMA3D* in c5 and c8. *TCF21* and *WT1* were widely expressed in the epicardial cluster except c8 (Fig. 3E). Cells expressing different gene sets exhibit varying differentiation tendencies. In the early stages of mouse and chicken embryos, *SCX*^+^ cell populations tend to differentiate to the endocardium, while *SEMA3D*^+^ cells contribute to the sinus venosus endothelium, and both of them participate in the formation of coronary vascular endothelium at later stages [44]. In zebrafish, co-expression of *TBX18*,* ACTA2*,* MYLKA*, and *SEMA3FB* promotes smooth muscle differentiation, whereas *TCF21*,* TBX18*,* WT1B*, and *TGM2B* maintain the integrity of the epicardium [45]. Different gene expression patterns may also indicate distinct developmental stages. During epicardium EMT, cell markers shift from *WT1*^*high*^*/TCF21*^*high*^ to *WT1*^*low*^*/TCF21*^*high*^. This suggests that gene expression differences do not reflect cellular heterogeneity but instead explain the transition of developmental states during ontogeny at some times [20]. Here, three genes (*UPK3B*,* SFRP5*,* SEMA3D*) were enriched in different clusters. High expression of *SEMA3D* may represent differentiation tendency to endothelium. While mesothelial gene *UPK3B* and embryonic epicardial gene *SFRP5* exhibit high expression in the epicardium of embryonic hearts at 9 PCW [41], and PEO has largely disappeared by 4 PCW, expression of these two genes may represent a developmental process, but the exact stage cannot be determined. *UPK3B* is widely expressed in cluster 4 at e18.5, whereas genes such as *WT1* and *TCF21* show high expression as early as e7.5. Combining time-course transcriptome and RNA velocity analysis, cluster 4 most likely represents a later developmental stage of epicardial development.

Specialization of the minority of posterior foregut or hepatic epithelium may be caused by the absence of RA during PE activation manipulated by WNT inhibition and BMP4 activation (d1.5 ~ d3.5) [46]. EC were also be validated by immunofluorescence showed in Fig. 3D and by TEM given the tight junction, adherens junction and ciliary tracts observed inside the organoid (Fig. 2F). The very few endodermal cells may be absent in some of epicardial organoids stained with AFP, CDX2 and HNF4A antibodies. Cluster 6, 9, 10 and 11 showed highly expression of proliferative markers including *TOP2A* and *MKI67* (Fig. 3D). Cluster 0, 1, 2 and 3 were classified as epicardium-derived cells (EPDCs) that undergoing EMT (*MOXD1*,* MMP11*,* TWIST1*,* SNAI1*,* ZEB2*). Markers indicating fibroblasts (FB) such as *LUM* and *TNC* as well as low CM marker (*HAND1*,* TNNT2*) expression were found in cluster 8 and 15. EPDCs could also give rise to mural cells marked by *PDGFRB* and *RGS5* (cluster 7). Cluster 4 and 16 exhibited an expression program alike to CM or SMC specific genes such as *PODXL*,* MYL7*,* RYR2*. The in vitro model also expressed several epicardial WNT signaling genes, such as *SFRP2*, *WNT2B* and *TFPI2*. The universal expression of human fetal epicardium genes (*SFRP2* [47], *SFRP5* [48], *COL9A3* [49], *TNNT1* [49], *TGB3* [50]) and angiogenic genes (*VEGFA*,* CXCL14* [51], *SLIT3* [49, 52]) implies epicardium of epicardial organoid resembles that of human fetal rather than adult heart epicardium. These features augment the regeneration ability of epicardium when used as the alternative way for heart healing [49].

C5 and c12 were extracted and re-clustered to obtain 9 subclusters (Fig. 3F). Among them, c8 co-expressed epicardium canonical markers (*WT1*,* TCF21*,* TBX18*,* SEMA3D*,* SCX*,* ALDH1A2*^*hi*^, *UPK3B*^*hi*^), but the expression level was moderate, and these cells accounted for only 0.7584% (36/4747). In addition to *TCF21* and *WT1*, the other epicardium markers may be inclined to repulsively expressed. These features verified the heterogeneity of epicardium which is associated with spatial cell location [45] and developing process [20]. There is controversy as to whether heterogeneity of epicardium originates from the PEO stage [20, 53]. Here, scRNA-seq results showed cellular differentiation of classical epicardium markers appeared at early timepoint, e.g. d7.5 and d9.5, that is, cellular heterogeneity exists in PEO, and PEO-derived epicardium carried this heterogeneity. Cell number of subclasses c3, c5, and c8 plunged at d18.5, and instead significantly increased with *CXCL14*^*hi*^*IGFBP3*^*hi*^*SFRP2*^*hi*^ cell populations which are the members of IGF and WNT signalling pathways (Supplementary Fig. 2C-E). WT1 was also found in PE [45] and is the positive upstream regulator of WNT signaling [54]. The expression of WT1 in PE may positively promote WNT activation, which feeds back into the specialization of the epicardium. To explore the propensity of transcription factors (TFs) in the process of cell fate restriction, we compared the TFs in EPDC (CM diff.), EPDC (FB & CM diff.) and Epi (EMT). *UPK3B*^*+*^ epicardium with high expressions of *EMX1*,* MAF*,* ELF4* and *NKX3-1* were more likely to differentiate into CMs (Fig. 3G, Supplementary Fig. 2F) while further functional experiments are needed for verification. Differential gene sets unveil the heterogenous spatial expression in epicardium that can serve as a cornerstone for inducing varieties of cell types.

### Epicardium integrated with cardiomyocyte both in vitro and in vivo

**Fig. 4:**
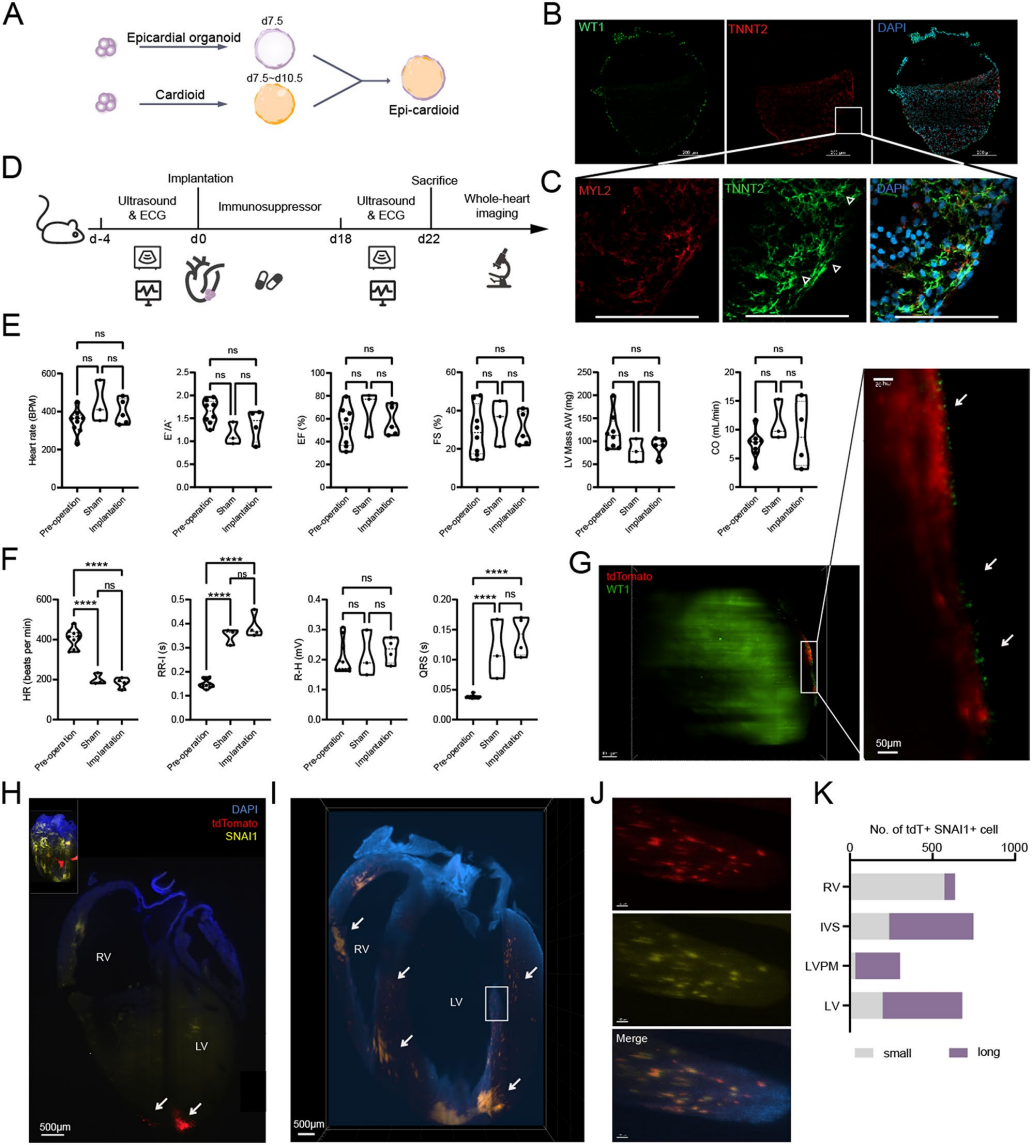
Epicardium exerts migration and EMT features both in vitro and in vivo. (**A**) Scheme of coculture of epicardium organoid and cardioid. (**B**) Epicardium wrapped outside the myocardium after coculturing epicardial organoid with cardioid for more than 1 month. (**C**) Enlarged image from white box of (**B**) showed sarcomere (arrowhead) and CM maturation marker MYL2. Scale bar, 100 μm. (**D**) Implantation procedure of epicardial organoid. Mouse heart function detected by ultrasonic test from parasternal long-axis view (PLAX), parasternal short-axis view (PSAX) and apical four-chamber view (AFC) (**E**), as well as electrocardiograph (ECG) test (**F**) before and after transplantation. Whole-heart staining showed human epicardial organoid integrated with mouse heart with WT1 + cells lining the most outside (**G**) and tdTomato + SNAI1 + cells spreading LV, RV, IVS and LVPM (**H**) and (**I**). (**J**) Enlarged picture of LVPM showed colocation of tdTomato + and SNAI1 + cells. (**K**) Cell number of tdT^+^ human cells distributed different parts of mouse heart. EF, ejection fraction. FS, fractional shortening. CO, cardiac output. HR, heart rate. RR-I, RR interval. R-H, R peak height. LV, left ventricle. RV, right ventricle. IVS, interventricular septum. LVPM, left ventricle papillary muscle. Significance analysis uses one-way ANOVA with the Tukey’s or Dunnett’s post-test correction was applied when appropriate (*p < 0.05, **p < 0.01, ***p < 0.001)

To investigate the migration characteristics of epicardial organoids when co-culturing with cardiac organoids, epicardium or epicardial organoids (d7.5 ~ d10.5) were co-cultured with cardiac organoids (d7.5, d12.5, d17.5) for more than 1 month until the fusion completes. The time points were chosen based on single-cell transcriptomic data, during which is the most similar stage with that of PEO migration in vivo. As shown in Fig. 4A and B, single layer of WT1^+^ cells lined outside multiple layers of TNNT2^+^ cells after one month co-culturing of d7.5 cardiac and epicardial organoids which resembles the real heart architecture. The long spindle shape of epicardium nuclei, as well as high expression of MYL2 in this assembly suggested the maturation of epicardium and cardiomyocyte (Fig. 4B and C) which may benefit from paracrine factors enriched in the expanded chamber. While co-culturing with epicardium single cells, there was regional expression of WT1^+^ and TNNT2^+^ cells, and the epicardium could not embed the CMs (Supplementary Fig. 3A). Both digested epicardium and the cells in the epicardial organoids went through a migration event while co-culturing with cardiac organoids, similar to the process of PEO onto the cardiac tube. In another word, the cells of this epicardial organoid have the ability of self-migration supports as a promising platform when co-culturing with cardiac organoids.

To interrogate the integrity efficiency and migration capability in vivo, epicardial organoid from day 7.5 to day 10.5 was transplanted into the subepicardium of mouse left ventricle. The experimental procedure is shown in the Fig. 4D. Due to the potential ethical issues caused by the use of hESCs in vivo, and the need of tracing the transplanted organoids, we introduced fluorescence protein with strong signal intensity that is tdTomato into hiPSCs. Sadly, fluorescence signal was not strong enough to penetrate sternum and skin when using IVIS Spectrum (PerkinElmer, Inc.). The introduction of tdTomato into iPSC cell line did not hamper the differentiation and formation of epicardial organoid (Supplementary Fig. 3B). Either the thoracotomy or the implantation of epicardial organoids did not affect the main heart function using ultrasonic technology as shown in Fig. 4E and Supplementary Fig. 3C. The calculated differences between pre-operation, sham and implantation groups were not significant on the aspect of ejection fraction (EF), fractional shortening (FS) and cardiac output (CO) ranging from 20% ~ 50%, 50% ~ 80% and 3% ~ 16%, respectively, while greater individual variability was observed with CO index. Hence, the ratio of E` and A` were all larger than 1. Cardiac function was also examined more subtly by ECG. There were significant differences in heart rate (HR), RR-interval (RR-I) and QRS duration between the pre-operation group and the sham group or the transplantation group. Thoracotomy may explain the stronger sensitivity to anesthetic isoflurane, resulting in a decrease in heart rate and an increase in RR-I without affecting the height of the R wave (R-H), which was 0.21 ± 0.08 mV in group sham and 0.23 ± 0.04 mV in group transplantation (Fig. 4F, Supplementary Fig. 3D). The increase in QRS may be the leading cause of the increase in RR-I. Fortunately, no differences in these parameters between the sham and the transplanted group were observed, suggesting that the side effect on mouse cardiac function was mainly due to thoracotomy rather than transplantation of hPSCs-derived epicardial organoid.

After 3-weeks recovery, mouse hearts were harvested for more tests. Organoids survived and integrated with mouse heart (Fig. 4H). WT1^+^ cells inside the organoid were found to be distributed along the outer edge of the heart (Fig. 4G). The migration event of proepicardium-derived cells translocating from proepicardium organ towards the atrioventricular canal of the nascent heart starts from E9.5 [55, 56] and Hamburger-Hamilton stage (HH) 17-18 [57] to E10.5 [56] in mouse and HH23 [58] in chick, respectively. In this model, there were numerous tdTomato positive cells translocating from the organoid entity. These cells were observed colocalizing with the EMT marker SNAI1 distributing in left ventricle (LV), right ventricle (RV), LV papillary muscle (LVPM), interventricular septum (IVS) regions (Fig. 4I). SNAI1^+^tdT^+^ cells in the basal LV and RV were small, while being large spindle-like vary from 90 μm to 170 μm in the apex of RV and IVS. Taking the location and cell size together, long SNAI1^+^tdT^+^ cells called for the potential of EPDCs differentiation to CMs-like cells. There were 271 long spindle-like SNAI1^+^tdT^+^ cells occupying the majority in LVPM showing a certain regular arrangement (Fig. 4J and K). Given that EMT genes were absent in human adult epicardium [49], this translocation and transition provide strong evidence for the resemblance of epicardial organoid to human fetal heart epicardium with high performance of migration and EMT.

## Discussion

The human epicardial organoids we established recapitulate two crucial features resemble fetal epicardium: generating other cell type by inducing EMT, and enlarging with subepicardium-like compartment abundant with ECM and GF. Epicardium in vivo provides diversified paracrine factors such as RA, TGF-β, BMP and collagens, driving CMs compaction and maturation, vessels formation, as well as structural and electrophysiological properties maintenance [59, 60]. This epicardial organoid supplied with some factors resemble that in vivo. However, the absent of CM may seal some functions of epicardium. Co-cultured organoids can be helpful for digging out more information about the interaction between epicardium and myocardium, such as the IGF2/IGF1R and NRP2 signaling discovered by Alessandra Moretti’s group [12]. Here, the assembloid of epicardial organoids and cardioids exhibited the tissue structure with high levels of *MYL2* and many sarcomeres that similar to human heart. We expect to explore more on the reactivation program under a milieu by co-culturing with cardiac organoids. The single-cell transcriptomics defined epicardial organoids as heterogeneous population at different developmental stages. The transcription factors *SCX* and *SEMA3D* in murine proepicardial cells were validated for prone to generating EC and coronary endothelium, respectively [44]. According to the interpretation of time-course transcriptome data, this heterogeneity of epicardium may stem from PE, scilicet cell fate is confined before EMT occurs.

Emerging evidence showed potential application of versatile epicardium on injured heart. For example, dissected human EPDCs remodeled infarcted mouse heart [61, 62], hPSCs-induced epicardium and CMs hosted vascularization of a ischemia/reperfusion rat heart [63], hPSCs-induced epicardium healed infarcted mouse and porcine heart by inhibiting inflammation through paracrine [15]. Epicardial progenitors are known to detach from the outer layer and undergo EMT to invade into subepicardium where other cell types can be further induced by both intrinsic and extrinsic signals [64]. EMT often takes place in 3rd trimester in fetal stage or been induced by heart injury such as ischemia and infarction after birth [19, 33, 34]. To identify those epicardial progenitors reactivate epicardium during heart regeneration is one of alternative strategies. Expression of WT1 requires recruitment of SWI/SNF subunit BRG1 by C/EBPβ onto conserved elements of WT1 and can be upregulated by interaction of BRG1 with thymosin-β4 [65]. The addition of thymosin-β4 was proved to change WT1^+^ cells into ISL1^+^NKX2-5^+^ cells which would differentiate into CMs with time limited before MI [13]. Studies identified substantial difference on cellular markers between embryonic mouse epicardium with adult mouse epicardium after heart injury [66]. Also in zebrafish, the heart regeneration only partially replicates developmental processes even for the same transcripts [67]. By implanting epicardial organoid into immunodeficient mice heart, we found that epicardium migrated to be attached the heart, and to the inner parts such as LVPM and IVS colocalizing with EMT marker *SNAI1* showing a CMs-like morphology. Fibroblast is another well-studied cell on transdifferentiation into CMs. However, this procession is absent under normal physiological conditions, and implementation requires human intervention, such as the canonical cocktail of *Gata4*,* Mef2c* and *Tbx5* [68]. Moreover, EPDCs (FB & CM diff.) only account for 2.83% of total from d7.5 and d9.5 epicardial organoids. The large number of CMs-like cells may be caused by spontaneous transdifferentiation of epicardial cells rather than fibroblasts. Even though, the potential effects of EPDCs (FB & CM diff.) from transplanted organoids should be considered. Origin of these CMs-like cells can be detected by introducing *CreER* after the promoters of epicardium and fibroblast specific markers as well as *lox*P-Stop-*lox*p to cardiomyocyte specific marker. If fluorescence inside the mouse hearts can be monitored in vivo by Biospace Optima small animal imaging system, path of organoid migration and CMs generation can be drawn out to interrogate the regional preference. By comparing the spatiotemporal transcriptome of the transplant assembloid with the single-cell transcriptome of organoids, potential pioneers with myocardial differentiation propensity could be identified, and verified on the aspect of transformation efficiency of indigenous cells in vitro and in vivo. The discovery of pioneer genes could reduce the inevitable trauma of surgery. Given the higher proportion of cardiomyocyte-like cells transformed from epicardium than in previous studies [15, 16], we expect its potential on transplantation or epicardial transdifferentiation for the treatment of cardiac injury.

## Conclusion

In summary, our results indicated that this epicardial organoid resembles human fetal-like epicardium on the aspect of EMT and paracrine ability as well as single cell transcriptome. Moreover, epicardial organoids in organoid fusion platform or transplantation system in vivo exhibited rapid migration performance and gave rise to CM-like cells with high efficiency.

## Electronic supplementary material

Below is the link to the electronic supplementary material.

Supplementary Material 1

Supplementary Material 2

## Data Availability

The data supporting this study’s findings are available from the corresponding author upon reasonable request.

## Abbreviations

PEO: pro-epicardium organ
E: embryonic day
SMC: smooth muscle cell
EMT: epithelial-mesenchymal transition
MI: myocardial infarction
CM: cardiomyocyte
hPSCs: human pluripotent stem cells
hESCs: Human embryonic stem cells
RA: Retinoic acid
VEGF: vascular endothelial growth factor
scRNA-seq: single-cell RNA sequencing
ZO-1: zonula occludens-1
TEM: transmission electron microscopy
GF: growth factor
FGF: fibroblast growth factor
EC: endocardium
pF/H Epithe: posterior foregut or hepatic epithelium
EPDCs: epicardium-derived cells
TF: transcriptional factor
ECG: electrocardiograph
EF: ejection fraction
FS: fractional shortening
CO: cardiac output
HR: heart rate
RR-I: RR-interval
HH: Hamburger-Hamilton stage
LV: left ventricle
RV: right ventricle
LVPM: left ventricle papillary muscle
IVS: interventricular septum
