# Supplementary material for: Human epicardial organoids from pluripotent stem cells resemble fetal stage with potential cardiomyocyte- transdifferentiation
Source: Cell Biosci. 2025 Jan 17;15:4. doi: 10.1186/s13578-024-01339-w (PMC11740338; doi:10.1186/s13578-024-01339-w)
Supplement: Supplementary file 2 — Supplementary Material 2 [file 13578_2024_1339_MOESM2_ESM.docx]

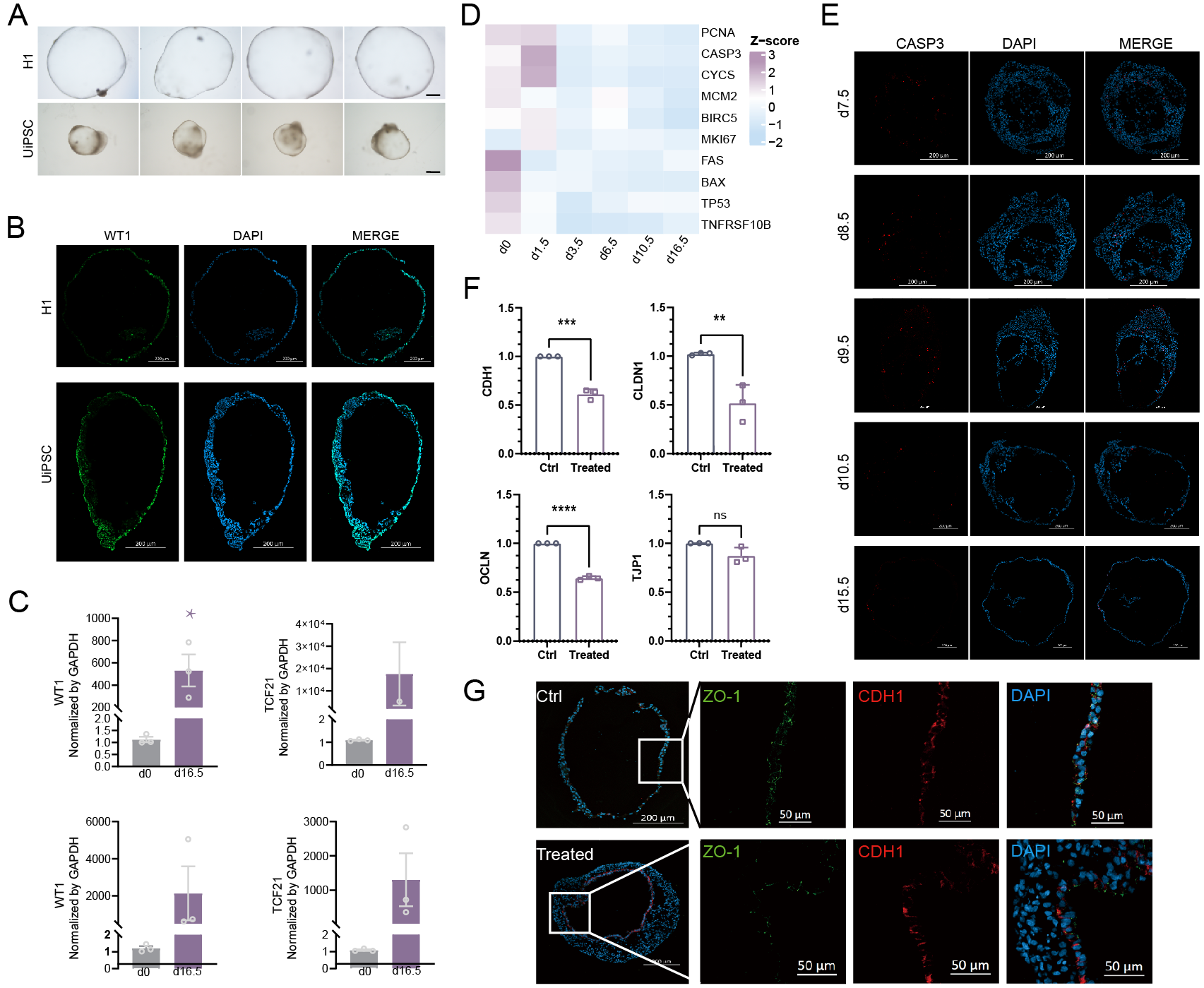


**Supplementary Fig. 1. Reproducibility of epicardial organoid from other hPSCs and apoptosis alongside induction timeline.** (A) Bright field images captured at d20 with four replicates each of H1 and home-made UiPSC exhibits great repeatability. Scale bar, 500 μm. (B) Immunofluorescence of epicardium marker WT1 at d20. (C) Quantification qRT-PCR analysis on epicardium markers *WT1* and *TCF21* at d16.5 of H1-derived organoid (upper) and UiPSC-derived organoid (lower). (D) Representative genes level of apoptosis extract from bulk RNA seq. (E) Apoptosis marker CASP3 protein level in organoid from day 7.5 to day 11.5. (F) mRNA level of tight junction (*TJP1, CLDN1, OCLN*) and adherens junction (*CDH1*) at d17.5 normalized by GAPDH. Ctrl, no EMT inducer, Treated, with EMT inducer. (G) Immunofluorescence of junction markers ZO-1 (*TJP1*) and CDH1 with or without EMT inducer. Significance analysis uses a standard unpaired Student t-test (2-tailed; *p < 0.05, **p < 0.01, ***p < 0.001). Images in a are representative of four independent differentiations.


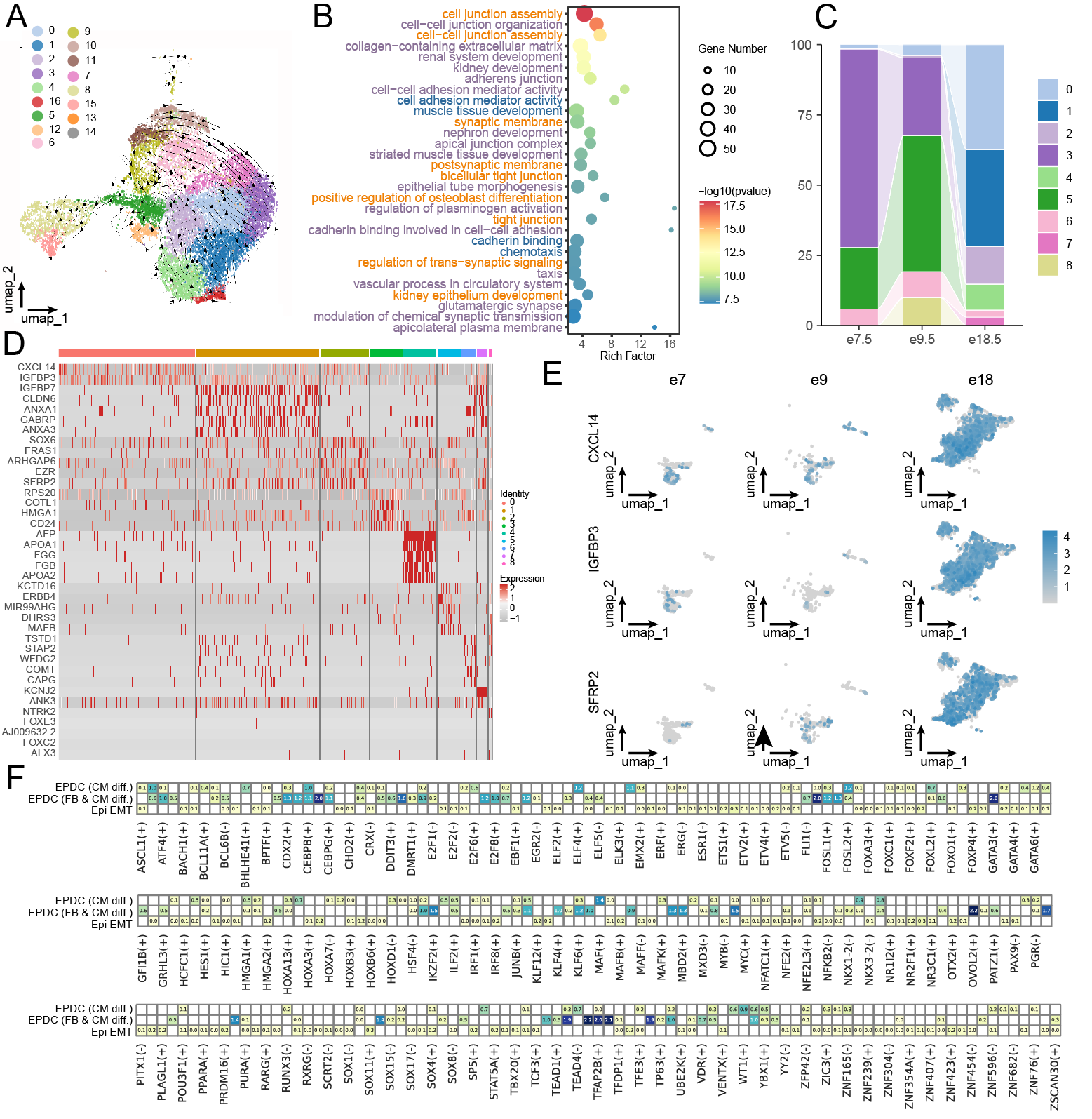


**Supplementary Fig. 2. Epicardial organoid with inner heterogeneity.** (A) RNA velocity analysis of single-cell transcriptome of epicardium organoid. (B) GO and KEGG signal enrichment analysis. (C) Stream flow plot for cell number percent of different subcluster. (D) Heatmap of key differential genes between 9 subclusters. (E) Feature plot of *CXCL4, IGFBP3* and *SFRP2* separated by time points. (F) Differential TF sets between EPDC (CM diff.), EPDC (CM diff.) and Epi (EMT).


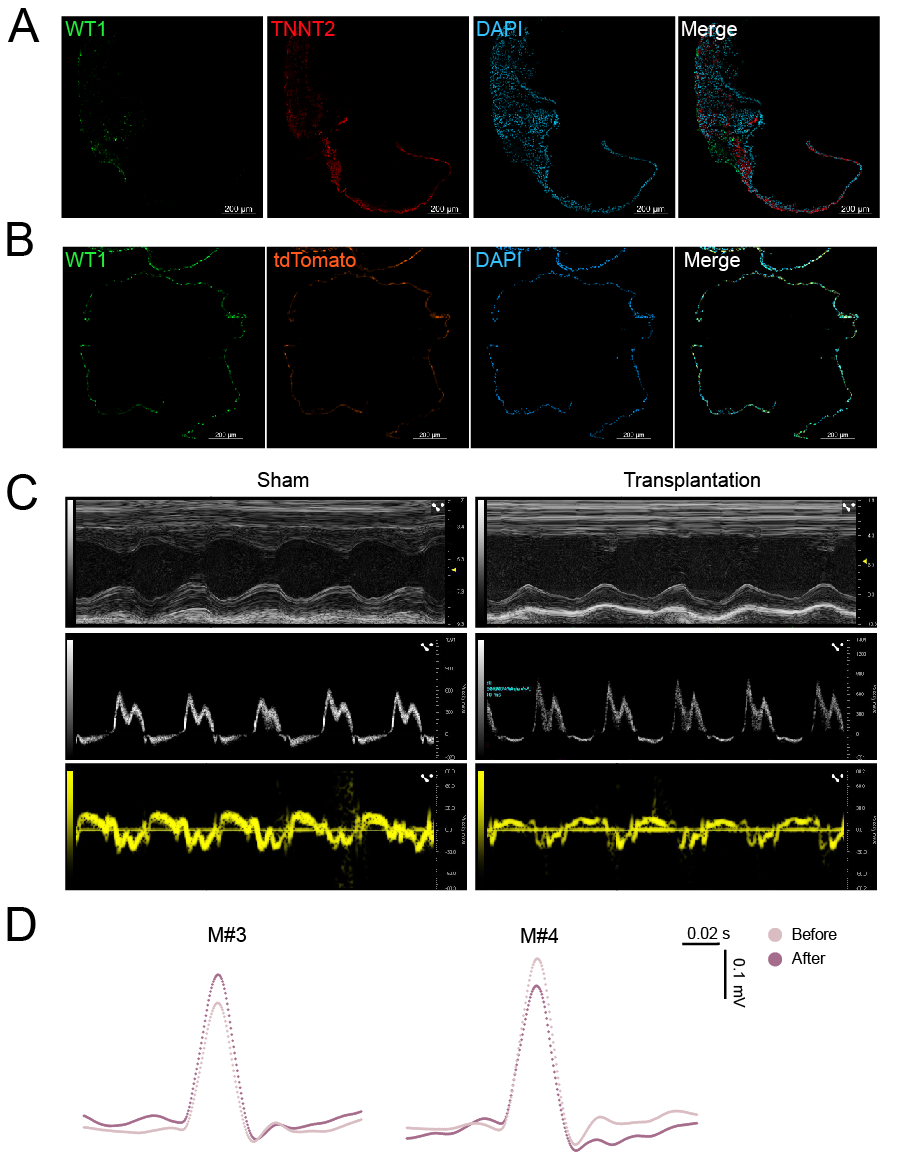


**Supplementary Fig. 3. Migration feature of epicardium.** (A) Fusion of epicardium cells with cardioids. (B) Expression of WT1 in organoid differentiated from tdTomato-iPSC. (C) Ultrasonic images from PLAX, PSAX and AFC view. (D) Identical ECG waveform plot before and after transplantation.
